# Prenatal PBDEs and Neurodevelopment: Accuracy of Assessment

**DOI:** 10.1289/ehp.1002748

**Published:** 2010-11

**Authors:** Julie E. Goodman, Giffe T. Johnson, Raymond D. Harbison, Richard V. Lee, Milo F. Pulde, Marcia Hardy, Todd Stedeford

**Affiliations:** Gradient, Cambridge, Massachusetts, E-mail: jgoodman@gradientcorp.com; University of South Florida, Tampa, Florida; State University of New York, Buffalo, New York; Harvard University Boston Massachusetts; Albemarle Corporation, Baton Rouge, Louisiana

Herbstman et al. (2010) measured eight polybrominated diphenyl ethers (PBDEs) in cord blood and reported that children of mothers with higher cord blood concentrations of PBDEs 47, 99, and 100 scored lower on mental and physical development tests at 12, 24, 36, and 72 months of age. Here, we raise several issues that limit the conclusions that may be drawn from their study.

In the study by Herbstman et al. (2010), only 210 cord blood specimens from 329 mothers were available, and assessments were conducted for only 96–118 children at each age. Several congeners were measured in the study; overall, the percentage of individual congeners below the limit of detection (LOD) ranged from 18.6% to 96.1%. For congeners on which major assessments were conducted, the range of values < LOD was 18.6–50.2%. Herbstman et al. (2010) did not state how many samples were < LOD for each assessment, so it is possible that the percentage was even higher and may have led to a large impact on the results, particularly given the small sample size for each assessment.

Herbstman et al. (2010) measured PBDEs in cord blood and maternal blood only once, but individual levels most likely changed over the course of the pregnancy and over the period when developmental assessments were conducted. The median values were relatively low, and there was no reliable indication of interindividual variability, so even small changes over time could have altered any observed exposure–response associations. In addition, the authors did not indicate how assessments of individual children changed over time. If different children contributed to associations at different ages, the reported findings may not be indicative of a causal role for PBDEs. Notably, most associations were found at earlier ages, suggesting that even if PBDEs were causal, effects were reversible.

Herbstman et al. (2010) reported that all PBDEs were correlated with one another, as were repeated developmental scores. Yet associations varied both among PBDEs for the same tests and among repeated tests for the same PBDEs. If PBDEs are truly causal and acting via similar mechanisms of action, results should be repeatable across PBDEs and repeated tests. This is not the case, thus suggesting that chance or some other factor (e.g., alcohol, caffeine, poor diet, methylmercury, polychlorinated biphenyls) is a more likely explanation. In addition, all of the mothers in the study were pregnant and lived near the World Trade Center (WTC) on 11 September 2001. Given this proximity, there is no way of knowing whether other unmeasured exposures or other factors (e.g., psychological, behavioral) contributed to neurological effects.

Herbstman et al. (2010) used the Bayley Scales of Infant Development, Second Edition (BSID-II) to measure developmental impairment, which is based on a mean ± SD of 100 ± 15 to define normal development (Bayley 1993). This means that in 68% of the standard population, scores ranged from 85 to 115. The authors reported that through univariate analysis, the change in the BSID-II score from the 25th percentile of PBDE level to the 75th percentile was −5.57. This degree of change is well within the SD of the test, which makes it impossible to determine whether the relationship observed was due to PBDEs or simply the interindividual variability inherent to the test. Because the authors did not assess the association between PBDE levels and those scoring outside the SD of the test (compared with those scoring within), it is impossible to determine whether a clinically significant association between PBDE cord blood levels and developmental impairment exists. Changes in IQ scores are not very meaningful unless they are put directly into context with the scoring ranges in the test design; Herbstman et al. did not provide much information as to the scores that were actually produced, though they implied that those from mothers with higher PBDE levels were somehow impaired when they may well have been normal.

Taken together, these factors prevent an accurate assessment of whether prenatal exposure to PBDEs is associated with adverse neurodevelopmental effects.
